# Regulatory T cells in human pregnancy: mechanisms, dysregulation, and therapeutic perspectives

**DOI:** 10.3389/fimmu.2026.1815957

**Published:** 2026-04-15

**Authors:** Huimin Liu, Wanrong Huang, Zhengyan Hu, Jinbiao Han, Rui Gao, Lang Qin

**Affiliations:** 1 Reproductive Medical Center, Department of Obstetrics and Gynecology, West China Second University Hospital, Sichuan University, Chengdu, China; 2 Key Laboratory of Birth Defects and Related Diseases of Women and Children, Ministry of Education, West China Second University Hospital, Sichuan University, Chengdu, China; 3 Department of Laboratory Medicine, West China Second University Hospital, Sichuan University, Chengdu, China; 4 Department of Obstetrics and Gynecology, West China Second University Hospital, Sichuan University, Chengdu, China; 5 Meishan City Maternal and Child Health Hospital, Meishan, China

**Keywords:** regulatory T cells, maternal-fetal immune tolerance, immunotherapy, recurrent implantation failure, recurrent spontaneous abortion, preeclampsia, preterm birth

## Abstract

Regulatory T cells (Tregs) play a central role in maintaining immune tolerance and supporting maternal-fetal homeostasis throughout human pregnancy. Clinical and experimental evidence demonstrates that dysregulation of peripheral and decidual Tregs manifested as quantitative deficits, impaired suppressive function or lineage instability increased the risk of various pathological pregnancies, such as recurrent implantation failure (RIF), recurrent spontaneous abortion (RSA), pre-eclampsia (PE) and preterm birth (PTB). Recently, novel therapeutic strategies targeting Tregs have emerged in oncology, transplantation, and autoimmune diseases. However, their application in pathological pregnancy remains in its infancy. This review outlines the spatiotemporal dynamics of peripheral and decidual Tregs throughout gestation, elucidating their roles in maintaining maternal-fetal homeostasis and their dysregulation in pathological pregnancies. We also critically evaluated the therapeutic strategies targeting Tregs and Tregs-associated signaling pathways, including hormonal support, traditional intravenous immunoglobulin, as well as emerging interventions such as immunometabolic reprogramming and engineered cellular therapies like chimeric antigen receptor Tregs. This review may provide insights for understanding the roles of Tregs in physiological and pathological pregnancy, as well as provide new idea in the immunotherapy of pathological pregnancy.

## 1 Introduction

Tregs are a specialized CD4^+^ T cell lineage essential for immune tolerance. The concept of CD4^+^CD25^+^ regulatory T cells was first established by Sakaguchi and colleagues (1). Subsequently, landmark studies identified forkhead box P3 (FOXP3) as a key lineage-defining transcription factor for Tregs, with later work further confirming its crucial role in human regulatory T cells (2–4). Although FOXP3 is the commonly used marker for canonical Tregs in pregnancy studies, FOXP3 expression alone does not fully define regulatory identity in humans (5, 6). Tregs are essential for autoimmune homeostasis in various organs and physiological processes. In pregnancy, the maternal immune system must tolerate the semi-allogeneic fetus carrying paternal antigens while simultaneously maintaining robust defense against pathogens (7, 8). Tregs form a central component of this adaptation and are now regarded as important mediators in establishing and maintaining maternal-fetal immune tolerance (9, 10). Our current understanding of Treg biology in pregnancy is informed not only by human studies but also by animal models, particularly murine models, which have provided key mechanistic evidence for the roles of Tregs in implantation, maternal-fetal tolerance, and pregnancy maintenance. However, species-specific differences should be taken into account when interpreting these findings in the context of human pregnancy.

Tregs populations are heterogeneous, comprising distinct subsets based on their origin and functional state (11). Thymus-derived Tregs (tTregs) circulate systemically to maintain tolerance to self-antigens (1), whereas peripherally induced Tregs (pTregs) differentiate from naive T cells upon antigen exposure in peripheral tissues and provide adaptive local immune suppression (11, 12). In addition, induced Tregs (iTregs) can be generated ex vivo under defined tolerogenic conditions such as TGF-β and IL-2 (13). However, especially in humans, no single marker has been universally accepted to unequivocally distinguish all of these subsets. In parallel, human FOXP3^+^CD4^+^ T cells are also commonly subdivided into resting, activated, and FOXP3^low^ non-suppressive populations based on activation or functional status (14). Evidence from other species, including rat, equine, bovine, and nonhuman primate models, has further expanded the framework of pregnancy-associated Treg biology and broadly supports their role in implantation, fetal tolerance, and pregnancy maintenance (15–18). However, species-specific differences in placentation and immune organization should be considered when translating these findings to human pregnancy.

At the maternal-fetal interface, a highly specialized and abundant population of decidual Tregs (dTregs) actively accumulates (19–21). These dTregs assembled through peripheral recruitment and local amplification are primed by endometrial stromal cells, trophoblast-derived antigens (e.g., HLA-G), and a unique local cytokine milieu (e.g., IL-10, TGF-β). Consequently, they are uniquely adapted to maintaining maternal-fetal immune tolerance and ensure placental development (9, 22). Furthermore, an emerging concept in both systemic immunology and pregnancy is the role of memory Tregs (mTregs), which provide long-term, antigen-specific immunosuppressive capacity (23, 24). This mTregs population may facilitate more rapid and robust tolerance in subsequent pregnancies, potentially reducing the risk of complications (25, 26). This capacity for immunological adaptation highlights a key parallel between pregnancy and other states of sustained immune modulation, such as chronic infection or cancer, where mTregs also play a pivotal role in regulating the immune response (27–29).

Despite significant progress, many studies examine only one aspect of Tregs biology, such as a single subset or gestational time point. Other work focuses on either physiological tolerance or pathological disruption without integrating both perspectives. There remains a need for a framework that links Treg dynamics across gestation with disease mechanisms and potential therapeutic strategies. This review elucidates the regulation of Tregs across gestation and links their dysfunction to complications including RIF, RSA, PE, and PTB. Furthermore, we evaluate current and emerging therapeutic strategies targeting the Treg axis to guide future clinical interventions.

## 2 Literature search

A literature search was conducted using PubMed, Web of Science and Scopus to identify studies relevant to the role of Tregs in physiological and pathological pregnancy. The search covered studies published from 1995 to 2025. Keywords included regulatory T cells, immune tolerance, maternal-fetal interface, implantation, decidua, pregnancy, recurrent implantation failure, recurrent miscarriage, preterm birth and pre-eclampsia. Additional terms were used to capture specific mechanistic themes such as endocrine regulation, cytokine signalling, immune checkpoints, metabolic adaptation and cell based immunotherapy. Reference lists of relevant publications were screened to identify additional studies. Studies were selected based on their relevance to the development, function, or dysregulation of Tregs in pregnancy, including human studies, animal models, and mechanistic *in vitro* work. When multiple publications reported similar findings, preference was given to more recent or more comprehensive studies.

## 3 Tregs in physiological pregnancy

Physiological pregnancy requires precise regulation of maternal immunity. A defining prerequisite for durable Treg function is epigenetic stabilization of the FOXP3 locus, particularly demethylation of the conserved non-coding sequence 2 (CNS2) or the Treg-specific demethylated region (TSDR), which ensures heritable FOXP3 expression and lineage stability across gestation (30).

On this stable molecular foundation, Tregs undergo dynamic changes in number, phenotype, and suppressive activity throughout pregnancy. These adaptations are shaped by integrated endocrine, cytokine, immune checkpoint, and metabolic cues. Together, these signals guide Tregs expansion, recruitment and functional specialization in a way that fits the needs of each stage of pregnancy.

### 3.1 Peri-implantation: orchestrating the “window of receptivity”

Implantation represents the initial critical juncture where the semi-allogeneic blastocyst challenges maternal immunity. Successful implantation depends on sufficient receptivity and appropriate responsiveness in the endometrium, as well as a developmentally competent blastocyst. During the mid-secretory phase of the endometrium, a transient “window of implantation” is formed, which permits embryo attachment and invasion. This process relies on the coordinated interaction among endometrial epithelial cells, stromal cells, and immune cells (31). Recent transcriptomic and single-cell sequencing studies have illuminated the critical importance of the immune cellular network in establishing endometrial receptivity. A significant number of differentially expressed genes between the early and mid-secretory phases are identified as immune or inflammatory regulators (32). Furthermore, single-cell analyses reveal that uterine immune cells undergo dynamic transcriptional changes during the mid-secretory phase (33), indicating that immunological adaptations for pregnancy commence even before embryonic arrival. These cells interact with non-immune cells in the epithelium, stroma, and vasculature, as well as with the embryonic trophoblast, profoundly influencing every stage of the implantation cascade. Among these, Tregs are multifaceted players, essential for establishing a “pro-gestational” state and initiating pregnancy through their roles in epithelial-embryo attachment, decidual transformation, trophoblast invasion, uterine vascular adaptation, and immune tolerance (34, 35).

The establishment of a tolerogenic microenvironment initiates prior to fertilization. First, seminal fluid drives immune priming. Exposure to paternal alloantigens together with TGF-β and prostaglandin E2 enriched in seminal plasma (36) can promote the initial expansion of paternal antigen-specific Tregs in the draining lymph nodes (37, 38). Consistent with this concept, Tsuda et al. reported clonal expansion of effector Tregs (CD4^+^CD25^+^CD45RA^−^FOXP3^high^) at the maternal-fetal interface and an association with pregnancy complications, indirectly supporting the establishment of antigen-specific tolerance prior to implantation (39). Nevertheless, direct confirmation of this hypothesis will require further evidence, such as pre-implantation TCR sequencing or antigen identification. Evidence from bovine artificial insemination and assisted reproduction settings suggests that this effect may not be uniform across all species and reproductive contexts (40). In women undergoing *in vitro* fertilization (IVF), seminal plasma exposure around oocyte pickup or embryo transfer has been associated with higher clinical pregnancy rates, but not with consistent improvement in ongoing pregnancy or live birth (41, 42), and a recent randomized trial likewise found no increase in clinical pregnancy or live birth after intravaginal seminal plasma exposure following oocyte pickup (43). Since successful pregnancies can also occur after artificial insemination or IVF without direct seminal exposure, seminal plasma is more likely to function as a tolerance enhancer. In such settings, other pathways of maternal immune adaptation are likely to compensate, although their relative contribution remains unclear.

**Figure 1:**
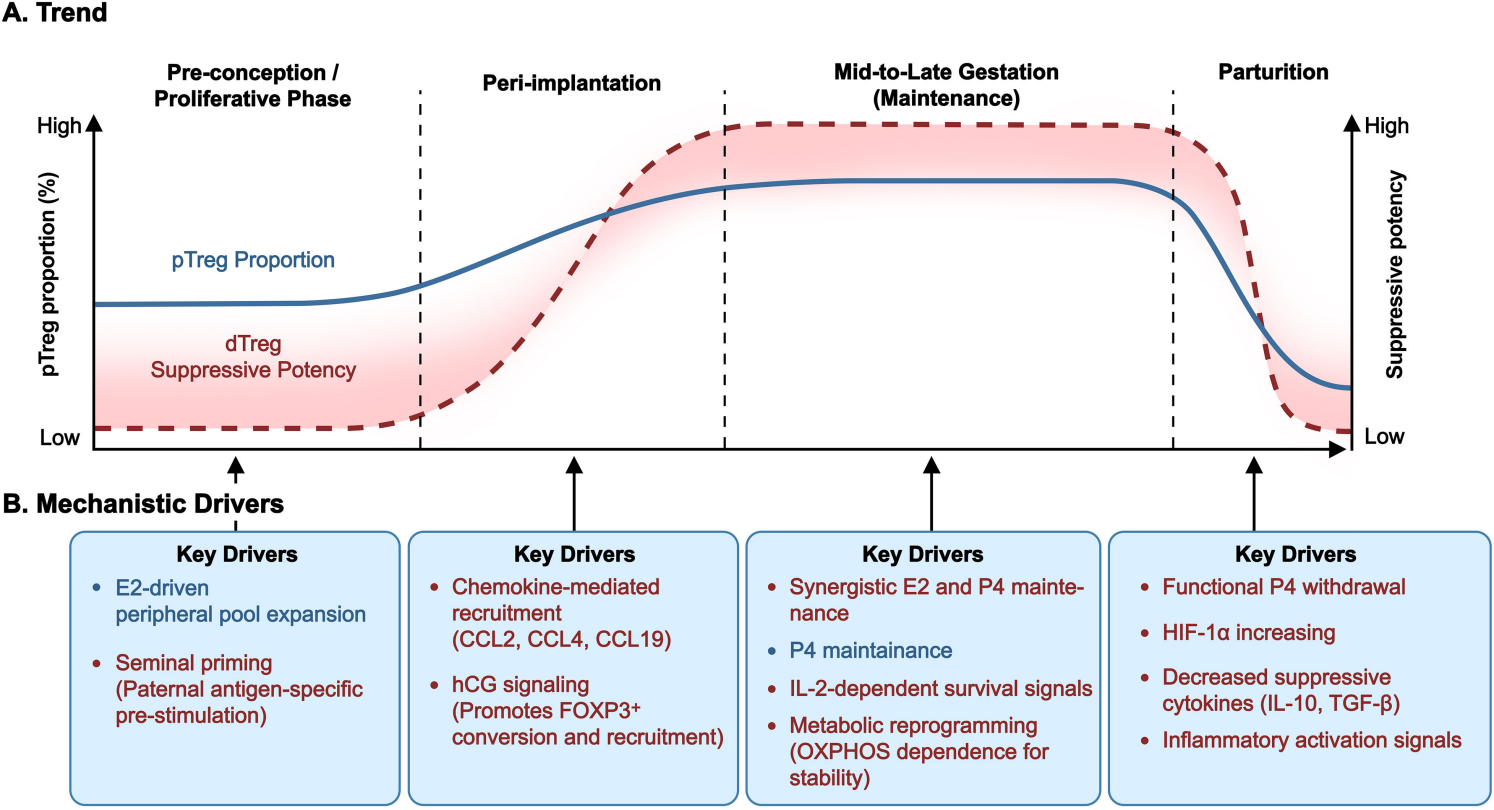
Spatiotemporal dynamics and functional determinants of Tregs across gestation. The upper **(A)** illustrates the trend of peripheral blood Treg proportions (Curve A) and the suppressive potency of dTregs (Curve B). While peripheral counts peak during mid-gestation, decidual functional activity is characterized by rapid induction during implantation and a critical collapse at parturition initiation. The lower **(B)** identifies the stage-specific hormonal, chemokine, and metabolic determinants that govern Treg recruitment, phenotypic conversion, and functional stability. CCL2/4/19, C-C motif chemokine ligand 2/4/19; dTreg, decidual regulatory T cell; E2, estradiol; FOXP3, forkhead box P3; hCG, human chorionic gonadotropin; HIF-1α, hypoxia-inducible factor 1-α; IL-2/10, interleukin-2/10; OXPHOS, oxidative phosphorylation; P4, progesterone; pTreg, peripheral regulatory T cell; TGF-β, transforming growth factor β.

Preparatory Treg accumulation from pTregs begins during the proliferative phase, driven by estrogen, and peaks at ovulation during menstruation (44). Following conception, dTreg enrichment is established early at the maternal-fetal interface (19, 20). In parallel, pTreg frequencies typically increase during early gestation, peak around mid-pregnancy, and decline prior to parturition (45, 46) (**Figure 1**). This antigen-driven expansion is powerfully augmented by a tolerogenic endocrine milieu. Pregnancy-associated hormones orchestrate a systemic and local surge in Treg numbers. Estrogen initiates preparatory recruitment of the pTreg pool during the late follicular and ovulatory phase (47–49). Subsequently, progesterone maintains elevated Treg proportions both systemically and locally within the decidual environment (50). In contrast, human chorionic gonadotropin (hCG) orchestrate the active recruitment of Tregs from the peripheral circulation and promote their decidua-specific accumulation and phenotypic conversion by inducing chemokine CCL2 (51–53). Concurrently, chemokines like CCL19 and CCL4 are essential for their active recruitment and localization during the peri-implantation period (54–56). It is noteworthy that in multiparous women, mTregs generated in previous gestations act as a rapid response force. They are recruited and reactivated immediately upon antigen re-encounter, thereby facilitating a more robust and efficient tolerance induction during the initial implantation window (24, 26).

Once localized at the implantation site, Tregs deploy sophisticated suppressive mechanisms. This includes direct, contact-dependent inhibition of effector T cells (Teffs). Tregs competitively inhibit CD28 co-stimulation by binding CD80/CD86 with high affinity, thereby blocking Teff activation (57–59). They also express a range of immune checkpoints, including cytotoxic T lymphocyte antigen 4 (CTLA-4), programmed cell death protein 1(PD-1), mucin-domain containing-3 (Tim-3), and lymphocyte-activation gene 3 (LAG-3), to further suppress Teff activity (60). Furthermore, Tregs express high levels of CD25, allowing them to sequester local IL-2. This deprives Teffs of essential survival signals, driving them toward apoptosis (61, 62). Beyond this direct suppression, Tregs function as critical immunological coordinators. They secrete cytokines like TGF-β to maintain their own Foxp3 expression (63) and stability and IL-10 to suppress local Th17-mediated inflammation (64, 65). They also modulate the phenotype of other local immune cells, promoting the differentiation of tolerogenic dendritic cells (tDCs) and M2-polarized macrophages via TGF-β, IL-10, and CTLA-4 signaling (66, 67). Moreover, Tregs inhibit decidual natural killer (dNK) cell proliferation and cytotoxicity by down regulating DC-mediated IL-15 release (68, 69).

This immune tolerance is not unilateral. There is a dynamic dialogue between Tregs and the invading trophoblast. Extravillous trophoblasts (EVTs) protect themselves from maternal Teffs and NK cell cytotoxicity by expressing the HLA-C, HLA-E and HLA-G (45, 70). Critically, they also secrete IL-10 and TGF-β, which aid in Treg recruitment and differentiation (71, 72). IL-10, in turn, has been shown to upregulate HLA-G expression (73), establishing a robust positive feedback loop that stabilizes the tolerogenic microenvironment essential for successful implantation.

### 3.2 Mid-gestation: tolerance maintenance and vascular remodeling

During mid-gestation, the primary challenge shifts from establishing systemic tolerance to maintaining a stable yet adaptable suppressive environment amidst rapid placental expansion and maternal cardiovascular adaptation (9, 10). By this stage, the maternal-fetal interface has matured into a complex ecosystem where Tregs must navigate a metabolically demanding microenvironment while interacting with a dense network of immune cells. Consequently, Tregs not only preserve immune homeostasis and restrain inflammation but also actively orchestrate spiral artery remodeling to support the blood supply for placenta and fetus (9, 74, 75).

The maintenance of a robust dTreg population depends on a supportive multi-dimensional niche that integrates hormonal programming with essential survival signals. In human systems, pregnancy-level progesterone and estradiol have been shown to expand CD4^+^CD25^+^FOXP3^+^ Tregs, enhance their suppressive capacity, and skew T helper responses toward anti-inflammatory profiles, through both direct hormone receptor signaling on Tregs and indirect effects on antigen presenting cells (APCs) (76–78). In pregnancy-specific settings, direct evidence for hormonal regulation of Tregs at the maternal-fetal interface is derived largely from murine models. Mid-gestation physiological progesterone promotes both systemic and uterine accumulation of CD4^+^CD25^+^ Tregs, enhances IL-10-linked suppressive function, and supports an anti-inflammatory immune balance (50). Mechanistically, progesterone acts directly on Tregs via the nuclear progesterone receptor (nPR)^67^. Locally, early pregnancy decidua contains activated CD4^+^ T cells, including FOXP3^+^ Tregs, that express high levels of IL-2Rα/β, indicating reliance on IL-2-dependent survival signals (80–82). Genetic and functional studies show that disruption of IL-2 or IL-2R signaling destabilizes FOXP3, contracts the Treg pool, and breaks immune tolerance, whereas adequate IL-2 maintains Treg proliferation, survival, and suppressive function (61, 62, 83, 84). Mechanistically, experimental disruption of these pathways precipitates Treg collapse and fetal rejection, confirming that sufficient IL-2 signaling within the decidual microenvironment is strongly implicated in sustaining a competent Treg compartment during pregnancy (82, 85, 86). Complementing these survival cues, recent evidence suggests a specialized retention mechanism where dNK cells secrete CCL1 to recruit and sustain CCR8^+^ Tregs, thereby anchoring this suppressive population at the maternal-fetal interface during placental development (87).

Concomitantly, dTregs undergo qualitative specialization as pregnancy progresses. In humans, phenotypic and single-cell TCR-sequencing data show that CD3^+^CD4^+^CD45RA^-^CD25^+^CD127^low/–^ effector Tregs expand clonally within the decidua during late gestation, with some clonotypes detectable across successive pregnancies, consistent with fetal or paternal antigen–specific activation and local effector differentiation (39). In mice, a related population of CCR5^+^ “effector” Tregs preferentially accumulates in the gravid uterus in response to paternal alloantigens, highlighting an antigen-experienced, tissue-tropic Treg subset that is selectively recruited during pregnancy (55, 56). This effector Treg population conceptually overlaps with mTregs, which possess long-lived, antigen-specific suppressive capacity and can be rapidly reactivated upon re-encounter with the same antigen (23, 24). Pregnancy-focused studies further support that fetal antigen–specific mTregs and clonally expanded effector dTregs form a type of “immunological memory” to previous pregnancies (88, 89). These cells contribute to more rapid and robust maternal-fetal tolerance in multigravidae and help maintain the stability of the tolerogenic environment across gestation (74, 90).

Metabolic signalings are important for dTregs. During decidualization and early gestation, the decidual microenvironment undergoes pronounced metabolic specialization, including relative hypoxia and fluctuating nutrient delivery (91). This microenvironment drives metabolic reprogramming in stromal and immune cells, enhancing glucose uptake, lipid handling, and amino-acid metabolism (92, 93). Decidualizing stromal cells also display Warburg-like glycolysis with increased lactate production and active lactate shuttling, while trophoblast-derived lactate further shapes local immune differentiation (94–96). Within this metabolically demanding environment, Tregs must adapt to sustain suppressive function. Compared with glycolysis-dependent Teffs, Tregs preferentially utilize oxidative phosphorylation and fatty-acid oxidation to generate ATP and preserve FOXP3 stability (97, 98). Reviews and experimental studies further show that immune cells, including Tregs, undergo coordinated metabolic reprogramming at the maternal-fetal interface to maintain tolerance under conditions of hypoxia and elevated lactate (99, 100). When Tregs are forced toward excessive glycolysis or their mitochondrial fatty-acid oxidation is impaired, FOXP3 expression becomes unstable. Consequently, cells may convert into pro-inflammatory state, a shift that is particularly detrimental in metabolically stressed placental tissues (101, 102).

Supported by this metabolic resilience, dTregs are empowered to orchestrate the critical process of spiral artery remodeling. Physiological transformation of spiral arteries, characterized by EVT mediated arterial widening and dNK cell supported matrix remodeling, requires a permissive decidual immune environment (34, 103). Tregs contribute to this environment by restraining local Teffs activation and promoting tolerogenic differentiation of decidual APCs to facilitate EVT migration (67, 68, 104). Direct mechanistic evidence confirms that transient loss of Foxp3^+^ Tregs leads to reduced dNK cell maturation, defective spiral artery remodeling, and fetal growth restriction (75). Additional work demonstrates that perturbations in pathways essential for Treg stability, for example, IL-2/IL-2R signaling producing comparable deficits *in utero*-placental vascular adaptation and maternal hemodynamic regulation (61, 105). Collectively, these findings demonstrate that a failure in the Treg regulatory axis, whether through insufficient accumulation, metabolic instability, or compromised signaling, may contribute to pathological vascular remodeling and PE (106, 107).

Altogether, mid-gestation defines a phase in which Tregs must integrate endocrine, cytokine, antigenic, and metabolic cues to preserve a large and highly specialized decidual pool. These cells sustain maternal-fetal tolerance and coordinate immune-trophoblast interactions to support deep spiral artery remodeling. Thus, Treg biology during mid-gestation represents a central checkpoint that couples systemic maternal adaptation to local placental health and shapes the trajectory of pregnancy outcomes.

### 3.3 Parturition: inflammatory switch

Parturition represents the physiological resolution of maternal-fetal tolerance in which the uterus gradually transitions from a predominantly anti-inflammatory state to a pro-inflammatory phenotype essential for labor (108). This shift accompanies progressive fetal maturation and mechanical stretch of the uterus, reflecting coordinated changes in endocrine signaling, myometrial excitability, and a metabolic adaptation including increased glycolytic signalling (109). Within this transitional immune framework, Treg dominance is reduced, permitting the activation of parturition-associated inflammatory pathways (110). By attenuating their suppressive functions, they allow a spatially and temporally restricted inflammatory cascade to unfold at term, ensuring successful delivery while preserving systemic maternal immune homeostasis (108, 111, 112).

As Treg-mediated suppression gradually wanes, the cytokine milieu at the maternal-fetal interface undergoes a marked shift. The dominance of IL-10 and TGF-β is progressively replaced by increased production of IL-1β, IL-6, TNF-α and chemokines that recruit neutrophils, macrophages and Teffs into the cervix, decidua and myometrium (108, 113–115). This pro-inflammatory environment promotes cervical ripening, matrix remodeling and myometrial contractions.

This decline in Treg dominance appears to coincide with endocrine changes associated with functional progesterone withdrawal. From early to mid-gestation, high levels of progesterone promote Treg expansion and function (50, 116). However, near term, changes in PR isoform expression in the myometrium and fetal membranes limit PR-B-mediated signalling, leading to reduced progesterone responsiveness and functional progesterone withdrawal (117–119). Whether a similar receptor shift occurs within Tregs themselves remains unclear. In parallel, a recent study suggested that inflammatory cues at the chorion-decidua interface induce progesterone-inactivating enzymes AKR1C1 (20α-hydroxysteroid dehydrogenase) in decidual stromal cells, thereby reducing local progesterone availability despite sustained systemic hormone levels (120, 121). This emerging stromal mechanism may contribute to functional progesterone withdrawal at term and weaken progesterone-supported immune regulation, although direct evidence that AKR1C1 acts on Tregs is still lacking.

At the metabolic level, labor is accompanied by hypoxia stress and increased glycolytic signalling within uterine tissues, together with a marked rise in local lactate concentrations (122–124). Although direct evidence in human decidua remains limited, such a hypoxic and glycolysis-biased microenvironment may further weaken regulatory restraint at term (99). In particular, hypoxia-inducible factor 1 α (HIF-1α) functions as a metabolic sensor capable of destabilizing FOXP3 (125, 126) and constraining Treg fitness under glycolysis-favoring conditions, a process that contrasts with the reliance of Tregs on oxidative phosphorylation and fatty-acid oxidation for lineage stability (97, 98, 127). In parallel, labor is associated with reduced abundance of PD-1 in exhausted T cell phenotypes at the maternal-fetal interface, suggesting attenuation of inhibitory checkpoint tone as delivery approaches (128). Collectively, these metabolic and checkpoint changes may act as reinforcing layers downstream of endocrine withdrawal and inflammatory amplification, biasing the local immune milieu toward effector dominance during parturition.

**Figure 2:**
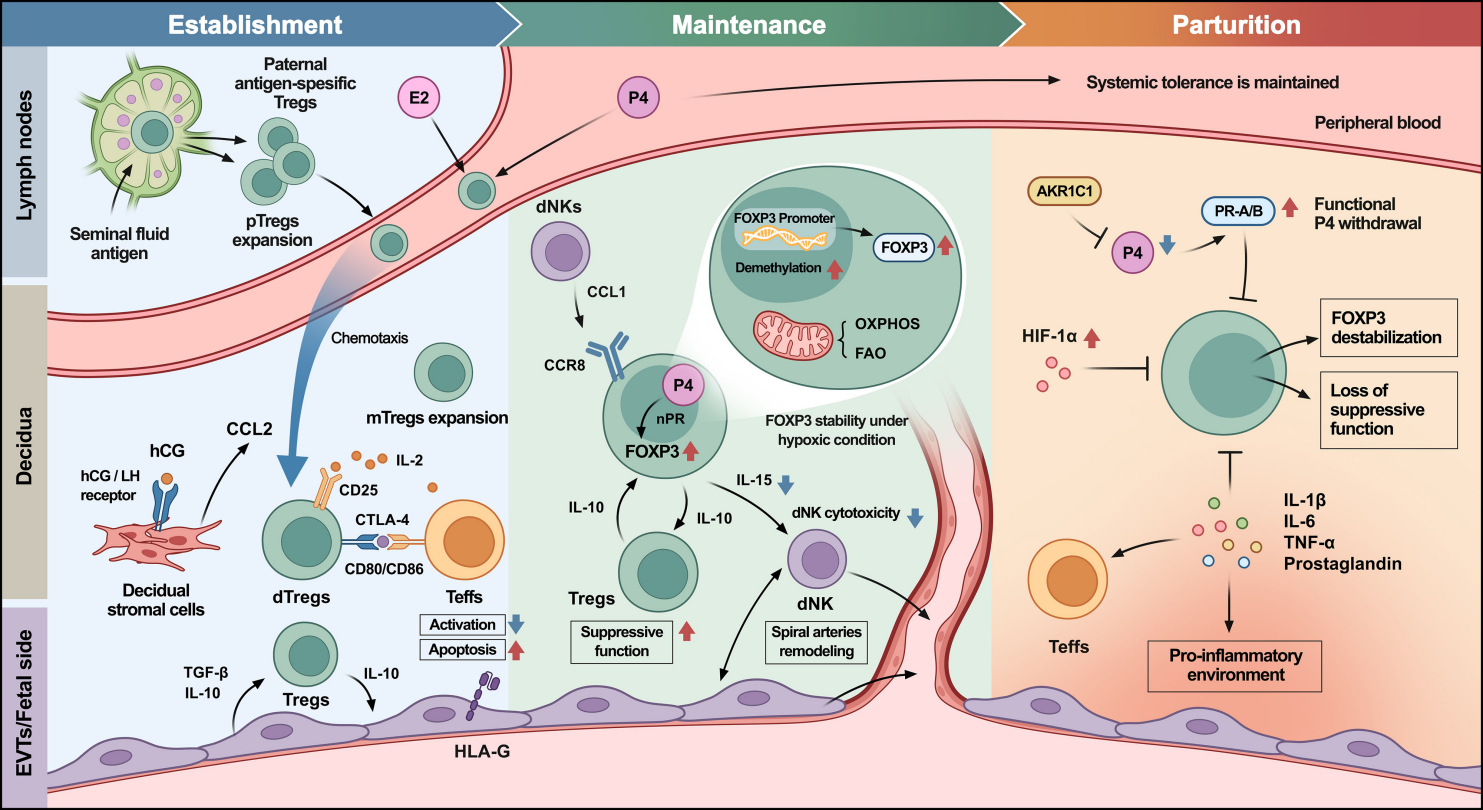
Molecular orchestrators of Treg-mediated maternal-fetal immune tolerance. This diagram illustrates the evolving molecular landscape of Tregs at the maternal-fetal interface. AKR1C1, aldo-keto reductase family 1 member C1; CCL1/2, C-C motif chemokine ligand1/2; CCR8, C-C motif chemokine receptor 8; CTLA-4, cytotoxic T-lymphocyte associated protein 4; dNK, decidual natural killer cell; E2, estradiol; EVT, extravillous trophoblast; FAO, fatty acid oxidation; FOXP3, forkhead box P3; hCG, human chorionic gonadotropin; HLA-G, human leukocyte antigen G; IL, interleukin; mTreg, memory regulatory T cell; OXPHOS, oxidative phosphorylation; P4, progesterone; PR-A/B, progesterone receptor isoform A/B; Teff, effector T cell; TGF-β, transforming growth factor β; TNF-α, tumor necrosis factor α; Treg, regulatory T cell. Some mechanisms shown are based on inference from prior literature and remain putative rather than directly demonstrated in the specific context discussed here.

Crucially, this inflammatory cascade remains tightly regulated. The persistence of clonally expanded effector Tregs in late gestation suggests that these cells continue to function as immunological barriers (39), confining inflammation to the reproductive tract (9, 22, 112). This ensures that while the uterus mounts an attack to deliver the fetus, systemic tolerance is preserved to protect the mother from generalized autoimmunity. Thus, Treg biology at parturition represents a sophisticated declination strategy rather than a total collapse of immunity (**Figure 2**).

## 4 Treg dysregulation in pathological pregnancy

Dysregulation of Tregs, including reduced numbers, impaired suppressive function and loss of lineage stability, is a common feature across pathological pregnancies. Clinical and experimental studies indicate that women with RIF, RSA, PTB and PE often show lower Treg frequencies or function in blood and decidua than women with healthy pregnancies (9, 22, 106). These quantitative deficits are frequently accompanied by a shift toward pro-inflammatory Th1 or Th17 immune microenvironment. A recent study further links RIF and RSA to a disrupted FOXP3 transcriptional programme and systemic Treg instability, suggesting that global Treg defects can underlie diverse obstetric complications (129). Furthermore, in infection-related pregnancy outcomes, the failure of Tregs to balance pathogen clearance with fetal tolerance represents a distinct axis of dysregulation. In this section, we summarize how Tregs abnormalities contribute to RIF, RSA, PTB, PE and infection-related pregnancy outcomes.

### 4.1 RIF

RIF is commonly defined as the inability to achieve a clinical pregnancy after the transfer of multiple high-quality embryos. In many cases, especially those involving euploid embryos, this condition reflects impaired endometrial receptivity driven by immune factors rather than intrinsic embryonic defects (31, 130, 131). Mid-secretory endometrium from women with RIF shows reduced FOXP3 expression and fewer FOXP3^+^ cells compared with fertile controls (132). Transcriptomic and single-cell analyses further highlight the importance of leukocyte regulation during the implantation window, showing that immune and inflammatory genes are the most dynamically regulated features during the implantation window (33, 133, 134). These molecular signatures suggest a close link between impaired endometrial immune adaptation and implantation failure, but do not clarify whether reduced Treg accumulation is a cause or a result of endometrial non-receptivity.

This loss of Treg dominance is consistently associated with a reciprocal shift toward Th17 responses. Systemically, women with RIF exhibit reduced frequencies of CD4^+^CD25^+^FOXP3^+^ Tregs and increased Th17 cells in the peripheral blood, resulting in a significantly elevated Th17/Treg ratio and signs of Treg exhaustion (135–137). Locally, in mid- and late-secretory endometrium, several studies report a depletion of FOXP3^+^ Tregs alongside elevated expression of pro-inflammatory cytokines such as IL-17, IL-6, TNF-α, and IL-8 (138, 139). This shift is likely to create an inflammatory milieu at the implantation site that antagonizes maternal tolerance and promotes embryo rejection. Several upstream mechanisms of hormonal guidance signals may also underlie this Treg paucity. Physiologically, hCG promotes Treg recruitment by stimulating endometrial stromal cells to produce CCL2 and by upregulating chemokine receptors such as CCR4 on Tregs. However, women with RIF may exhibit impaired activation of this recruitment axis, resulting in fewer endometrial Tregs at baseline (51, 52). The upstream basis of this impaired response remains unclear, but may involve reduced endometrial stromal responsiveness to embryonic hCG (140). This pathway should therefore be regarded as one potential component of the implantation-related immune network.

Together, these data support a close association between systemic Treg decline and impaired local immune adaptation, lowering threshold for embryo rejection. Occurring against a Th17-biased inflammatory background and compounded by defects in homing, this dysregulation may contribute to failed implantation, while a fundamentally non-receptive endometrium may likewise fail to support appropriate Treg recruitment and expansion.

### 4.2 RSA

The breakdown of immune tolerance at the maternal-fetal interface is intrinsically linked to RSA. Single-cell analyses of the decidual niche highlight that this tolerance failure involves a specific reduction in suppressive T cell states and disrupted crosstalk between T cells, NK cells, and macrophages (141–143).

Within this dysregulated environment, Treg abnormalities represent an important component of the failed immune regulation. Specifically, women with unexplained RSA show reduced frequencies of CD4^+^CD25^+^FOXP3^+^ Tregs in peripheral blood and decidua (144, 145), and low circulating Treg numbers in early pregnancy serve as a predictive marker for subsequent loss in high-risk women (146). Notably, compared with other pregnancy complications, RSA currently has relatively stronger subset-level evidence, suggesting that qualitative changes in specialized dTreg subsets, such as CCR8^+^ and Tim-3^+^ populations, may accompany the overall reduction in bulk FOXP3^+^ Tregs (87, 147). In addtion, these cells exhibit qualitative defects, as Tregs are selectively reduced and display impaired suppressive function in decidua tissues from miscarriage (148). Furthermore, RSA patients exhibit lower FOXP3 expression (145, 149) and diminished production of anti-inflammatory cytokines like IL-10 and TGF-β (150). This deficiency leads to a systemic and local shift toward Th1 and Th17 immunity, characterized by an increased Th17 to Treg ratio and elevated levels of IL-6 and IL-17 (151–153).

Several upstream mechanisms have been identified as drivers of these Treg abnormalities. At the mechanistic level, impaired Tregs function in unexplained RSA has been linked to aberrant Toll-like receptor 4 and NF-κB signaling (154). Epigenetic dysregulation constitutes another layer of repression. Clinical evidence reveals that the FOXP3 promoter region is significantly hypermethylated in the decidual tissues of RSA patients. This epigenetic inhibition shows a strong negative correlation with FOXP3 protein levels, thereby destabilizing the Treg lineage (155, 156). Additionally, reduced responsiveness to IL-2 and TGF-β limits FOXP3 induction and impede the expansion of fetal antigen specific Treg populations (82, 154, 157). Local microenvironmental factors may also contribute, as a recent study identified a CCR8^+^ dTreg subset and suggested that reduced CCL1^+^ dNK cells may impair the local accumulation or maintenance of these cells at the maternal-fetal interface (87). Experimental evidence from abortion prone mouse models confirms that Tregs are essential for fetal survival (158). In these models, the depletion of Tregs precipitates embryo resorption, whereas the adoptive transfer of Tregs restores tolerance and rescues the pregnancy (159–161).

Similarly, the defective generation or maintenance of fetal antigen specific mTregs may be a critical factor of RSA. Such impairment prevents the maternal immune system from mounting an adequate tolerogenic response in subsequent gestations, thereby predisposing women to repeated pregnancy losses despite successful implantation (26, 162).

### 4.3 PE

PE is a pregnancy-specific disorder characterized by new-onset hypertension and proteinuria, originating from impaired trophoblast invasion and defective spiral artery remodeling (163). Although the etiology is multifactorial, immunological maladaptation is considered a precipitating factor. Failure of the regulatory immune response may contribute to the shallow placentation and systemic endothelial dysfunction (164–166). Clinical studies consistently demonstrate that women with PE exhibit a profound deficit in the Tregs compartment compared with normotensive pregnancies, involving both quantitative reductions and qualitative functional impairments (167). Women with PE have lower percentages of Tregs in peripheral blood and decidua than healthy pregnant controls (168–171). Specifically, Toldi et al. showed that effector Tregs are preferentially reduced in PE, whereas naïve Tregs remain relatively preserved, indicating that the Treg defect in PE is subset-specific rather than a homogeneous global decline (172). Functionally, Tregs in PE also display reduced expression of anti-inflammatory mediators such as IL-10 and CTLA-4, leading to less efficient control of Teff proliferation. This breakdown in regulation is consistent with a reciprocal shift toward Th1- and Th17-dominated responses at the maternal-fetal interface. Consequently, the Treg/Th17 ratio is significantly reduced in PE and correlates with disease severity (106, 173, 174).

The deficiency of Treg mediated tolerance is likely to participate in vascular pathology of PE. In PE, Treg insufficiency may unleash cytotoxic NK cells and macrophages, which target invasive trophoblasts and impair their ability to remodel maternal vessels (105, 175). Furthermore, reduced Treg-derived IL-10 exacerbates this injury, as IL-10 is critical for maintaining vascular integrity and limiting the release of anti-angiogenic responses. Its deficiency in PE has been linked to accelerated endothelial dysfunction and hypertension (176, 177). Treg dysfunction can therefore be viewed as one upstream contributor that amplifies the cascade of placental ischemia and maternal vascular damage (174). Several upstream mechanisms have been proposed to underlie this Treg dysfunction. At the molecular level, one plausible contributor is the overexpression of soluble endoglin (sEng), a pseudoreceptor that acts as a decoy for TGF-β. High levels of sEng in PE patients are thought to sequester circulating TGF-β, thereby weakening a key signaling pathway involved in FOXP3 induction (178, 179). At the level of cellular crosstalk, defective interactions between Tregs and their stromal partners are evident. Decidual DC-SIGN^+^ APCs, which normally promote Treg induction, are numerically and functionally altered in PE, leading to loss of the close spatial association typically seen in normal placentas (180, 181). Additionally, checkpoint signaling is dysregulated. The PD-1/PD-L1 checkpoint pathway which normally promotes Treg differentiation and restrains Th17 responses is altered in women with PE (182). Moreover, PD-L1-Fc treatment in a rat model of PE ameliorates hypertension, proteinuria, and the Treg/Th17 imbalance (183). These abnormalities are further shaped by metabolic and epigenetic stressors. Given the hypoxic environment of the PE placenta, HIF-1α stabilization appears to favor Th17 differentiation while promoting FOXP3 degradation via the mTOR glycolysis axis, whereas oxidative phosphorylation and fatty-acid oxidation support Tregs lineage stability and suppressive function. This provides a plausible route by which established PE pathology can secondarily intensify Treg dysfunction (125, 184, 185). Overall, Treg dysregulation in PE is better understood as part of a self-reinforcing pathogenic loop.

Experimental models support a causal contribution of Treg deficiency to the pathological features of PE, particularly when Treg loss occurs early in gestation. Transient depletion of Tregs in Foxp3-DTR and related mouse models leads to impaired uterine artery adaptation, defective spiral artery remodeling, reduced uterine NK cell numbers, fetal growth restriction and late-gestation fetal loss, thereby recapitulating key aspects of the human disease (75, 105, 186). Conversely, adoptive transfer or pharmacological expansion of Tregs in PE-like rat models effectively ameliorates hypertension and restores uteroplacental vascular remodeling (15, 187). In these models, IL-10 deficiency further exacerbates disease severity, indicating that Tregs and IL-10 act together to mainta*in utero*-placental vascular integrity and maternal blood pressure control (177).

Evidence from human and animal studies suggests that Treg dysfunction is an important component of PE pathogenesis. Together with defective microenvironmental support, altered checkpoint signaling and metabolic stress, this Treg insufficiency likely compromises spiral artery remodeling and reinforces the anti-angiogenic environment that drives maternal endothelial damage.

### 4.4 PTB

PTB remains a leading cause of neonatal morbidity and mortality and is intimately associated with aberrant immune activation and premature inflammation at the maternal-fetal interface. Under physiological conditions, late pregnancy is sustained by a strong immunoregulatory environment that prevents untimely initiation of labor. Disruption of this regulatory balance lowers the inflammatory threshold required to activate parturition pathways and predisposes to PTB (188). Accumulating evidence indicates that PTB is not a single disease but rather reflects a state of dysregulated immune activation in which suppressive mechanisms fail to adequately inhibit pro-inflammatory responses.

A consistent immunological feature observed in PTB is the imbalance between Tregs and Teffs. Integrative analyses of decidual immune profiles show that women with PTB frequently exhibit reduced Treg frequencies or impaired suppressive capacity, accompanied by a skewing toward Th1- and Th17-dominant responses (189). These alterations resemble, but occur prematurely compared with, the immune shifts that normally precede term labor, suggesting an accelerated or dysregulated inflammatory trajectory. Clinical studies further support the notion that Treg abnormalities may precede the onset of spontaneous PTB and thus serve as a potential risk indicator. In a longitudinal study of maternal blood, CD4^+^CD25^+^FOXP3^+^ Tregs frequencies were highest in mid-pregnancy, declined at labor, and were lowest in women with spontaneous PTB complicated by inflammatory or infectious complications (110). In contrast, women destined for idiopathic spontaneous PTB exhibit a premature and more profound reduction in these cells as early as the first trimester (190). Beyond simple numerical deficits, the qualitative composition of Treg undergoes a pathological shift. Specifically, a reduction in highly suppressive HLA-DR^+^ Tregs occurred in women with term and preterm labor, suggesting a qualitative rather than purely quantitative defect (191). In addition, Gomez-Lopez et al. reported that functional Tregs are reduced at the maternal-fetal interface in a subset of women with idiopathic spontaneous PTB, further indicating that PTB-associated Treg insufficiency may involve not only circulating numerical decline but also local loss of suppressive Treg activity (192). Together, these findings support that Treg insufficiency is not merely a consequence of labor-associated inflammation, but may represent an upstream vulnerability factor in at least a subset of PTB cases.

A potential mechanism by which Treg deficiency may promote PTB involves the unchecked activation of both innate and adaptive inflammatory cascades. In PTB, CD4^+^CD25^+^FOXP3^+^ Tregs exhibit diminished ability to suppress Teffs proliferation and reduced control over Toll-like receptor-driven cytokine production by maternal leukocytes (111). The resulting increase in pro-inflammatory cytokines and prostaglandins promotes cervical ripening, membrane weakening, and myometrial contractility. Moreover, this inflammatory milieu is further amplified by the expansion of Tc17 cells (CD45^+^CD3^+^CD8^+^IL-17A^+^), which sustain T cell mediated placental inflammation (192). Experimental models provided causal evidence linking Treg loss to PTB. Partial depletion of FOXP3^+^ Tregs in late gestation mice precipitated PTB, fetal growth restriction, and neonatal mortality, whereas adoptive transfer of functional Tregs restores fetal survival and suppresses uterine inflammatory mediators (192). Furthermore, in models of intrauterine inflammation induced by low-dose lipopolysaccharide, Treg-deficient dams display markedly higher susceptibility to PTB, indicating that Tregs normally buffer inflammatory responses to pathogen-associated molecular patterns and tissue stress signals (193).

In infection-associated or sterile inflammatory forms of PTB, including acute chorioamnionitis, the initiating events differ from those of idiopathic PTB. In these settings, intra-amniotic infection or tissue injury constitutes the primary inflammatory stimulus, inducing the production of chemokines, cytokines, and prostaglandins by neutrophils, macrophages, and T cells within the chorioamniotic membranes and decidua. Rather than serving as the initial trigger, insufficient Treg-mediated regulation permits inadequate containment of this response, thereby facilitating a self-amplifying inflammatory cascade that promotes myometrial activation and membrane rupture, leading to PTB (194).

In summary, current evidence including preclinical models supports the possibility that early or excessive loss of Treg-mediated control represents one mechanistically relevant pathway in a subset of PTB cases. In this endotype, reduced systemic and dTreg numbers, and a shift away from highly suppressive Treg subsets allow innate and Teffs to respond more vigorously to microbial products and tissue damage signals, which contribute to initiation of PTB. However, the heterogeneity of findings across cohorts and the prominence of other immune pathways indicate that Treg dysfunction likely contributes to PTB in concert with broader disturbances of the decidual immune network rather than in isolation.

### 4.5 Infectious complications of pregnancy

Maternal infections are a common cause of adverse pregnancy outcomes, including miscarriage, spontaneous PTB, stillbirth, intrauterine fetal demise, congenital infection and neonatal sepsis (195–197). Acute infection can damage the placenta or trigger intrauterine inflammation, and vertical or perinatal transmission can lead to severe neonatal disease and death. Infection-related PTB is also linked with maternal sepsis, fetal death, neonatal sepsis and long-term neurodevelopmental impairment (198, 199). Against this background, pregnancy must balance immune tolerance to the fetus with antimicrobial defense. Tregs are central to maintaining this delicate equilibrium at the maternal-fetal interface (200). Consequently, the disruption of Treg homeostasis by infectious agents often dictates the clinical trajectory of the pregnancy.

Clinical observations reveal that different pathogens manipulate the Treg compartment in divergent ways, leading to distinct immunopathological states. Acute systemic infections, such as severe SARS-CoV-2, are characterized by a profound Treg/Th17 imbalance and systemic hyperinflammation. In these cases, the failure of regulatory control correlates with high rates of PTB and placental injury (201). Conversely, chronic or endemic infections may exploit the physiological expansion of Tregs to evade maternal clearance. In malaria-endemic regions, placental *Plasmodium falciparum* infection and *in utero*-antigen exposure are associated with increased frequencies of cord blood CD4^+^CD25^+^FOXP3^+^ Tregs and elevated IL-10 production (202). While this heightened prenatal tolerance may limit local tissue damage, it potentially hinders pathogen eradication and predisposes infants to increased infection susceptibility after birth.

Mechanistic insight from murine models of protozoal and bacterial infection. In *Toxoplasma gondii* (T. gondii) infection, excreted-secreted antigens (ESA) drive adverse pregnancy outcomes by directly compromising the decidual Treg pool. ESA triggers Treg apoptosis and destabilizes the lineage by suppressing FOXP3 transcription via the inhibition of TGF-β/Smad and IL-2R/STAT signaling, concurrent with the hyperactivation of the PI3K-AKT-mTOR axis. This functional collapse precipitates fetal loss, whereas ACT or IL-10 administration can rescue the tolerogenic microenvironment (29). In contrast, *Listeria monocytogenes* (Lm) induces fetal wastage through a distinct qualitative mechanism that operates even in the absence of direct placental invasion. Unlike the numerical depletion seen in toxoplasmosis, Lm preserves the size of the maternal Treg pool but critically blunts its suppressive potency. This functional paralysis appears to depend on the entry of bacteria into the host cell cytoplasm, a process that attenuates Treg-mediated suppression and unleashes the expansion of fetal-specific effector T cells. This leads to a state of sterile immune-mediated resorption, where the maternal immune system identifies the fetus as the primary target of an uncontrolled inflammatory response (203).

These preclinical data suggest that pathogen-specific disruption of Treg stability and function may represent one important mechanism in infection-related pregnancy disorders. While T. gondii physically depletes the tolerogenic pool via apoptosis, Lm functionally blunts suppressive potency to unleash anti-fetal immunity. Conversely, chronic pathogens may exploit physiological Treg expansion to evade clearance. Thus, the clinical outcome may be shaped by how the specific infectious agent manipulates the delicate tolerance-defense axis.

Where direct decidual evidence is limited, peripheral blood findings are discussed as supportive systemic correlates rather than direct surrogates of local maternal-fetal interface biology, and murine studies are cited mainly for mechanistic insight rather than as direct equivalents of human decidual Treg biology.

Although these conditions differ in clinical presentation, several mechanistic themes recur across them. These include reduced Treg abundance, impaired suppressive function, defective recruitment or retention, instability of the FOXP3 program, and a shift toward Th1/Th17-dominant inflammation. Disruption of IL-2, TGF-β, IL-10, immune checkpoint, and metabolic pathways appears to represent a shared upstream framework linking these disorders, as summarized in **Table 1**.

**Table 1:** Overview of Treg dysregulation and mechanisms in pathological pregnancies.

Complication	Treg characteristics (quantity and function)	Regulatory mechanisms	References
RIF	Quantitative deficit: Reduced circulating and endometrial FOXP3^+^ Treg frequencies. Functional exhaustion: Signs of phenotypic exhaustion and skewed Th17/Treg ratio. Recruitment failure: baseline paucity of endometrial Tregs.	Hormonal-chemokine axis defect: Impaired hCG signaling fails to stimulate decidual stromal cell production of CCL2 or upregulate CCR4 on Tregs. Inflammatory milieu: Local upregulation of pro-inflammatory cytokines (IL-6, IL-17) inhibits tolerance establishment.	(51, 52, 132, 135–139)
RSA	Quantitative deficit: Decreased Tregs in peripheral blood and decidua; specific loss of CCR8^+^ subsets. Functional impairment: Reduced IL-10/TGF-β production and FOXP3 expression. Differentiation failure: Defective generation of fetal antigen-specific mTregs.	Signaling aberrations and epigenetic dysregulation: Dysregulated TLR4/NF-κB signaling, reduced responsiveness to IL-2/TGF-β and aberrant DNA methylation at regulatory loci. Crosstalk disruption: Insufficient CCL1 production by dNK cells may impair CCR8^+^ Treg recruitment and retention. Expansion failure: Inability to mount rapid tolerogenic responses in subsequent pregnancies.	(26, 82, 87, 144–146, 148–157, 162)
PE	Quantitative deficit: Lower percentages of Tregs in peripheral blood and decidua. Qualitative defects: Downregulated CTLA-4 and IL-10 expression; reduced suppressive capacity against Teffs and innate cells. Th17 shift: Marked decrease in Treg/Th17 ratio.	Molecular decoy: sEng sequesters TGF-β, blocking FOXP3 induction. Metabolic reprogramming: HIF-1α accumulation promotes Th17 differentiation and FOXP3 degradation via the mTOR-glycolysis axis. Checkpoint dysregulation: Altered PD-1/PD-L1 signaling Stromal defects: Impaired interaction with DC-SIGN^+^ APCs.	(105, 106, 125, 155, 156, 168–171, 173–185)
PTB	Quantitative deficit: Accelerated reduction of circulating Tregs prior to labor onset (idiopathic PTB). Subset loss: Specific depletion of the highly suppressive HLA-DR^+^ phenotype. Compromised suppression: Inability to restrain Teff proliferation and Tc17 expansion.	Unchecked inflammation: Failure to buffer TLR-driven cytokine production and innate inflammatory cascades. Pathway activation: Loss of regulation lowers the threshold for pro-inflammatory pathways (prostaglandins, cytokines) that trigger myometrial activation.	(110, 111, 189–192)
Infectious Complications	Pathogen-specific dysregulation: *Malaria*: Pathological expansion and elevated IL-10. *T. gondii*: Numerical depletion via apoptosis. *L. monocytogenes*: Functional paralysis despite normal numbers.	Chronic infection: Exploitation of physiological tolerance mechanisms to evade immune clearance. T. gondii: ESA mediates inhibition of TGF-β/Smad signaling and and IL-2R/STAT signaling, concurrent with the hyperactivation of the PI3K-AKT-mTOR axis. L. monocytogenes: Intracellular bacterial entry directly blunts suppressive potency.	(29, 201–203)

AKT, protein kinase B; APC, antigen-presenting cell; CCL/CCR, C-C motif chemokine ligand/receptor; CTLA-4, cytotoxic T-lymphocyte associated protein 4; DC, dendritic cell; DC-SIGN, dendritic cell-specific intercellular adhesion molecule-3-grabbing non-integrin; dNK, decidual natural killer cell; ESA, excreted-secreted antigens; FOXP3, forkhead box P3; hCG, human chorionic gonadotropin; HIF-1α, hypoxia-inducible factor 1-α; HLA, human leukocyte antigen; IL, interleukin; mTOR, mammalian target of rapamycin; mTreg, memory regulatory T cell; NF-κB, nuclear factor kappa B; PD-1, programmed cell death protein 1; PD-L1, programmed death-ligand 1; PE, preeclampsia; PI3K, phosphoinositide 3-kinase; PTB, preterm birth; RIF, recurrent implantation failure; RSA, recurrent spontaneous abortion; sEng, soluble endoglin; STAT, signal transducer and activator of transcription; Tc17, cytotoxic T 17 cell; Teff, effector T cell; TGF-β, transforming growth factor beta; Th1/17, T helper 1/17 cell; TLR, toll-like receptor; TNF-α, tumor necrosis factor alpha; Treg, regulatory T cell.

## 5 Therapeutic modulation of the Treg axis

**Figure 3:**
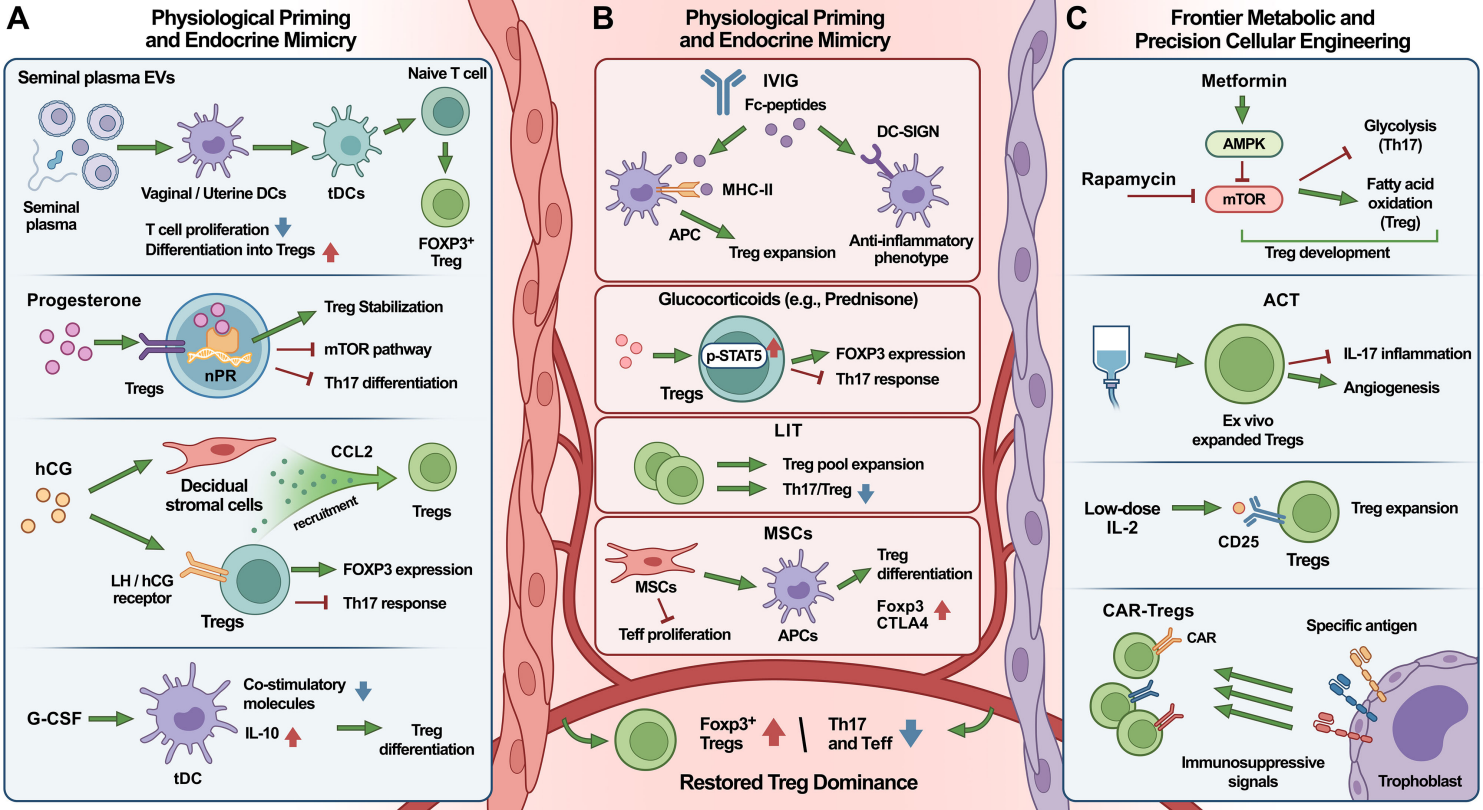
Therapeutic strategies targeting the Treg axis in pregnancy complications. This schematic outlines interventions designed to restore Treg dominance at the maternal-fetal interface. **(A)** Physiological priming and endocrine signals: seminal plasma EVs and G-CSF induce tDCs to promote Treg differentiation. Progesterone and hCG mimic natural tolerance by enhancing Treg stability and endometrial recruitment while inhibiting Th17 responses. **(B)** Clinical immunomodulation and cell therapies: IVIG, glucocorticoids, LIT, and MSCs reprogram APCs and suppress Teffs to expand the Treg pool. **(C)** Frontier metabolic and cellular engineering: metabolic modulators (metformin, mTOR inhibitors) shift cellular metabolism to favor Tregs over Th17 cells. Precision therapies, including low-dose IL-2, ACT, and CAR-Tregs, specifically reconstitute the tolerogenic microenvironment. AMPK, AMP-activated protein kinase; APC, antigen-presenting cell; CAR, chimeric antigen receptor; DC-SIGN, dendritic cell-specific intercellular adhesion molecule-3-grabbing non-integrin; EVs, extracellular vesicles; G-CSF, granulocyte colony-stimulating factor; hCG, human chorionic gonadotropin; IVIG, intravenous immunoglobulin; LIT, lymphocyte immunotherapy; MHC-II, major histocompatibility complex class II; MSCs, mesenchymal stem cells; mTOR, mammalian target of rapamycin; nPR, nuclear progesterone receptor; STAT5, signal transducer and activator of transcription 5; tDCs, tolerogenic dendritic cells.

Defects in the number or function of Tregs are a consistent feature of several pregnancy complications (106). Consequently, restoring Treg dominance has become a central therapeutic goal. Current strategies range from physiological priming and endocrine support to established clinical immunomodulation and emerging metabolic or cellular engineering. In addition to their therapeutic modality, these interventions may also be considered according to the dominant pattern of Treg dysfunction they are most likely to address, such as defective recruitment or maintenance, impaired suppressive function, Th17-skewed inflammation, or loss of Treg stability within inflammatory microenvironments. The following subsections summarize these approaches, focusing on interventions where mechanistic studies have demonstrated a direct restorative effect on the Treg axis (**Figure 3**).

### 5.1 Seminal fluid and hormonal priming

The most fundamental approach to therapeutic modulation involves reinforcing the natural signaling pathways that initiate maternal tolerance. Seminal plasma exposure represents one of the earliest physiological signals capable of shaping maternal immune adaptation. Seminal fluid may therefore be viewed as a context-dependent enhancer of tolerogenic conditioning, including the expansion or functional support of Treg-related pathways. Extracellular vesicles (EVs) within seminal plasma inhibit T cell proliferation and promote FOXP3 and IL-10 expression, and interact with vaginal and uterine DCs to induce a tolerogenic phenotype, which subsequently primes naive T cells toward a FOXP3-expressing regulatory phenotype (204, 205).

Following this initial priming, endocrine support maintains the Treg niche throughout gestation (10). Progesterone has been shown to shift the immune balance by downregulating Th1/Th17 cytokines while promoting anti-inflammatory mediators (206–208). Progesterone signaling via the nuclear progesterone receptor (nPR) appears to be important for the expansion of maternal Tregs (79). At the molecular level, progesterone stabilizes the Treg lineage by inhibiting the mTOR pathway and suppressing Th17 differentiation (76, 209). For hCG, systemic administration has been observed to increase endometrial Treg accumulation (52, 53) and reduce fetal resorption in abortion-prone models (210). Systemically, hCG promotes Treg homing to the endometrium by inducing stromal CCL2 secretion (51), while locally acting via LH/hCG receptors on Tregs to drive FOXP3 expression and restrict pro-inflammatory Th17 responses (211). Together, these endocrine signals help reinforce early tolerogenic conditioning and support local Treg recruitment and maintenance at the maternal-fetal interface.

Similarly, granulocyte colony-stimulating factor (G-CSF) has been linked to increased dTreg density in women with reproductive failure (212, 213). The underlying immunological mechanism involves the induction of tDCs, which downregulate costimulatory molecules and secrete IL-10 to prime the differentiation of naive T cells into Tregs (214). Despite these mechanistic insights, robust clinical evidence for G-CSF in reproductive failure remains lacking, and its routine use is not recommended (215).

### 5.2 Intravenous immunoglobulin, glucocorticoids, and alloantigen immunotherapy

In addition to physiological signaling, established clinical interventions are employed to recalibrate the maternal-fetal immune composition, particularly in settings marked by impaired suppressive Treg function or persistent pro-inflammatory skewing. Intravenous immunoglobulin (IVIG) exerts broad immunomodulatory effects through Fc-derived peptides that can promote Treg activation. These peptides are presented by MHC-II molecules to directly expand the Treg pool and reprogram APCs toward an anti-inflammatory phenotype via DC-SIGN receptors (216, 217). Clinical evidence suggests that high-dose IVIG can increase pTreg proportions and reduces NK cell activity, particularly in women with a history of recurrent losses and documented immune deviations (218–222).

Calcineurin inhibitors, specifically tacrolimus, have been explored in small cohorts of women with RIF and Th1 dominance (223, 224). While clinical studies report corrected Th1/Th2 ratios and improved implantation rates, direct evidence linking these outcomes to Treg expansion remains scarce. Additionally, TNF-α inhibitors target the pro-inflammatory cytokines often implicated in RSA (225). Observational series suggest that TNF-α blockade is associated with higher live birth rates in selected women (225, 226). Although TNF-α inhibitors are known to restore defective Treg function and expand FOXP3^+^ populations in autoimmune disorders (227, 228), longitudinal studies confirming this specific pathway in pregnant women are absent. Consequently, the presumed Treg-mediated benefit in adverse pregnancy outcomes remains largely an extrapolation from autoimmune diseases rather than a demonstrated mechanism in obstetric cohorts.

Glucocorticoids offer alternative systemic modulation. Low-dose prednisone has been reported to shift the Th17/Treg balance by upregulating STAT5 phosphorylation, which drives FOXP3 expression while simultaneously repressing the Th17 lineage (229, 230). Despite these mechanistic benefits, expert consensus advises caution, reserving glucocorticoid therapy strictly for patients with documented immune abnormalities, such as altered peripheral Th1/Th2 ratios or elevated uterine NK cell counts (231).

The use of cell-based therapies provides a more complex biological approach to resetting the tolerogenic environment. Paternal lymphocyte immunotherapy (LIT) mimics physiological alloantigen exposure to therapeutically reset the maternal Treg axis. Studies have shown that this treatment could correct Th17/Treg imbalances and expand the CD4^+^CD25^+^FOXP3^+^ Treg pool in women with unexplained RSA (232, 233), but its clinical application remains a subject of debate due to conflicting efficacy data. In parallel, mesenchymal stem cell (MSC) therapy offers a potent, albeit experimental, cellular approach. Derived from bone marrow, decidua, or amniotic tissues, MSCs possess the capacity to create an immune-privileged microenvironment. *In vitro*, MSCs inhibit Teff proliferation and modulate APCs to induce Treg differentiation (234). In abortion-prone murine models, MSC administration successfully reduces fetal loss and increases Treg density at the maternal-fetal interface by upregulating key regulatory genes such as Foxp3 and CTLA4 (235).

Collectively, these approaches are intended to restore suppressive Treg activity and rebalance persistent inflammatory antagonism.

### 5.3 Metformin, mTOR inhibitors, and precision cellular engineering

Immunometabolic modulation represents a novel frontier for influencing Treg fate, particularly in settings where Treg dysfunction is linked to metabolic stress or loss of lineage stability. This concept is based on the finding that Tregs rely more heavily on oxidative phosphorylation while Teffs depend primarily on glycolysis. Metformin is an insulin-sensitising drug that also affects immune metabolism. In women with repeated pregnancy loss, Fu et al. reported that metformin treatment restored the Th17/Treg balance by modifying STAT signalling and increasing FOXP3 expression, thereby improving pregnancy outcomes (236). Mechanistically, this effect involves the activation of 5’ adenosine monophosphate-activated protein kinase (AMPK), which in turn inhibits the mTORC1 pathway. This metabolic shift selectively represses the glycolysis required for Th17 differentiation while metabolically favoring Treg development, which relies on mitochondrial fatty acid oxidation (237). Similarly, the mTOR pathway serves as a central metabolic switch that, when hyperactivated, promotes Th17 mediated inflammation at the expense of Treg maintenance in RSA (238). Therapeutic targeting of this pathway has shown promise in preclinical models. Research by Royster and colleagues showed that treatment with the mTOR inhibitor rapamycin effectively corrected Treg deficits, thereby restoring implantation rates and normalizing litter sizes in mice susceptible to reproductive failure (239). These findings identify mTOR-related metabolic reprogramming as a potentially useful strategy when Treg insufficiency is coupled to inflammatory or metabolic imbalance. However, rapamycin (sirolimus) should still be regarded as experimental in pregnancy, because placental safety data are limited and current prescribing information and clinical guidance advise caution or avoidance during pregnancy due to potential fetal risk and insufficient human data (240).

Epigenetic regulation represents a fundamental layer governing Treg stability and suppressive durability. Preclinical studies show that histone deacetylase inhibition can reinforce FOXP3 expression and enhance Treg function ex vivo, enabling the generation of functionally optimized Tregs for adoptive transfer (161). In contrast, studies involving DNA methyltransferase inhibition highlight the essential role of intact epigenetic control in decidual and placental development, underscoring the need for careful contextualization (155, 241). These findings position epigenetic modulation primarily as a strategy for ex vivo Treg engineering rather than direct *in vivo* intervention during pregnancy.

Adoptive Treg transfer (ACT) and cytokine therapy offer a more direct approach to reconstitute the tolerogenic pool, particularly in conditions marked by profound Treg deficiency or failure of endogenous regulatory recovery. In classic murine abortion models, the transfer of pregnancy-induced Tregs reverses implantation failure and fetal resorption by suppressing IL-17-driven inflammation and promoting placental angiogenesis (159, 160, 242–244). Parallel strategies using low-dose IL-2, which selectively expands Tregs via high-affinity CD25 signaling, have also proven effective in reducing hypertension and mitochondrial dysfunction in PE models (245). Although clinical-grade Treg products and low-dose IL-2 are already in advanced trials for transplantation and autoimmunity, their application in human pregnancy remains a promising area for future clinical investigation.

Chimeric Antigen Receptor (CAR)-Tregs represent the vanguard of precision tolerance. Unlike polyclonal Tregs, CAR-Tregs are engineered to express a synthetic receptor targeting a specific antigen, guiding them to the tissue of interest. In transplantation models, CAR-Tregs demonstrate superior homing to antigen-expressing grafts, where they deliver potent, localized immunosuppression with minimal systemic side effects (246, 247). Although still theoretical in the context of pregnancy, CAR-Tregs could target trophoblast antigens to specifically restore maternal-fetal tolerance in severe, refractory pregnancy complications. Future efforts will need to focus on identifying suitable fetal targets and validating long-term safety before translation to high-risk pregnancies.

Overall, metabolic, epigenetic, and engineered cellular strategies are most relevant when Treg dysfunction extends beyond simple numerical loss to include instability, impaired fitness, or failure to remain suppressive within inflammatory microenvironments.

## 6 Discussion

Tregs are essential for establishing and maintaining maternal-fetal immune tolerance by integrating systemic endocrine cues with local microenvironmental signals to regulate embryo implantation, placental development, and parturition. Unlike the immune imbalance observed in autoimmune diseases or oncology, maternal-fetal tolerance depends on a dynamic balance between anti- and pro-inflammatory milieus that shifts across gestation, though the core mechanisms remain incompletely defined. Dysregulation of this axis through quantitative deficits, impaired suppressive function, or lineage instability lowers the threshold for pathological inflammation, driving diverse complications.

A major strength of this review lies in the integration of immunological, endocrine and microenvironmental perspectives to provide a unified framework for understanding Treg function across physiological and pathological pregnancy. However, an important limitation is that much of the mechanistic insight into Treg regulation has been derived from animal models or peripheral immune analyses, with comparatively limited direct investigation of human decidual and placental tissues. At the same time, findings across multiple animal species support the broad relevance of Tregs in pregnancy, but also highlight that differences in placentation, decidual architecture, and immune composition may constrain direct translation to human pregnancy.

Another important conceptual consideration is that FOXP3 expression alone does not necessarily identify suppressive Tregs in human tissues. Conversely, FOXP3-independent regulatory populations, such as Tr1 cells, may also contribute to immune tolerance at the maternal-fetal interface (5). Thus, while FOXP3-based phenotyping remains a practical and widely used framework in pregnancy research, it likely captures only part of the broader regulatory T-cell landscape. Future studies should combine phenotypic, functional, and epigenetic approaches to better define regulatory immune subsets in normal and pathological pregnancy.

Therapeutic strategies in this field are shifting from empirical immunomodulation toward precision approaches that target defined regulatory pathways. Advances in immunometabolic modulation, epigenetic conditioning, and cellular engineering provide complementary tools to generate functionally stable and context-adapted Tregs, with epigenetic modulation applied ex vivo to reinforce FOXP3 stability and suppressive durability. In parallel, effective clinical translation requires patient stratification rather than uniform application of immune-based therapies. Given the marked heterogeneity of pregnancy complications, future efforts should prioritize clinical entities with limited treatment options and clearer immunological involvement, such as unexplained RSA.

Further progress requires establishing causal relationships between Treg alterations and pregnancy outcomes while exploring regulatory mechanisms to identify precise therapeutic targets with minimal systemic interference. Integrating Treg metrics with clinical phenotypes to develop risk prediction models will help identify high-risk populations most likely to benefit from immune intervention. Given the physiological and ethical constraints of pregnancy, new therapies must undergo rigorous evaluation of safety for both mother and offspring. Collectively, these advances support the development of targeted interventions for pathological pregnancies.
